# Self-Reinforced Composite Materials: Frictional Analysis and Its Implications for Prosthetic Socket Design

**DOI:** 10.3390/ma17225629

**Published:** 2024-11-18

**Authors:** Yogeshvaran R. Nagarajan, Yasasween Hewavidana, Emrah Demirci, Yong Sun, Farukh Farukh, Karthikeyan Kandan

**Affiliations:** 1 School of Engineering and Sustainable Development, De Montfort University, The Gateway, Leicester LE1 9BH, UK; yogeshvaran.nagarajan@my365.dmu.ac.uk (Y.R.N.); ysun01@dmu.ac.uk (Y.S.); f.farukh@dmu.ac.uk (F.F.); 2 Composite Research Group, University of Nottingham, Nottingham NG7 2GX, UK; 3 Wolfson School of Engineering & Sustainable Development, Loughborough University, Loughborough LE11 3TU, UK; y.hewavidana@lboro.ac.uk (Y.H.); e.demirci@lboro.ac.uk (E.D.); 4 Faculty of Engineering, Universitas Negeri Padang, Padang 25131, Indonesia

**Keywords:** prosthetic socket, self-reinforced polymer, friction and wear, sustainable composites

## Abstract

Friction and wear characteristics play a critical role in the functionality and durability of prosthetic sockets, which are essential components in lower-limb prostheses. Traditionally, these sockets are manufactured from bulk polymers or composite materials reinforced with advanced carbon, glass, and Kevlar fibres. However, issues of accessibility, affordability, and sustainability remain, particularly in less-resourced regions. This study investigates the potential of self-reinforced polymer composites (SRPCs), including poly-lactic acid (PLA), polyethylene terephthalate (PET), glass fibre (GF), and carbon fibre (CF), as sustainable alternatives for socket manufacturing. The tribological behaviour of these self-reinforced polymers (SrPs) was evaluated through experimental friction tests, comparing their performance to commonly used materials like high-density polyethylene (HDPE) and polypropylene (PP). Under varying loads and rotational speeds, HDPE and PP exhibited lower coefficients of friction (COF) compared to SrPLA, SrPET, SrGF, and SrCF. SrPLA recorded the highest average COF of 0.45 at 5 N and 240 rpm, while SrPET demonstrated the lowest COF of 0.15 under the same conditions. Microscopic analysis revealed significant variations in wear depth, with SrPLA showing the most profound wear, followed by SrCF, SrGF, and SrPET. In all cases, debris from the reinforcement adhered to the steel ball surface, influencing the COF. While these findings are based on friction tests against steel, they provide valuable insights into the durability and wear resistance of SRPCs, a crucial consideration for socket applications. This study highlights the importance of tribological analysis for optimising prosthetic socket design, contributing to enhanced functionality and comfort for amputees. Further research, including friction testing with skin-contact scenarios, is necessary to fully understand the implications of these materials in real-world prosthetic applications.

## 1. Introduction

The global population of amputees is steadily increasing, driven by factors such as rising chronic health conditions and accidents [1,2]. This growing demographic underscores the critical need for advanced prosthetic devices to restore mobility and improve the quality of life for individuals with limb loss [3,4]. Among these devices, the prosthetic socket is a fundamental component connecting the artificial limb to the residual limb, ensuring functional stability and user comfort [5,6]. Therefore, the design of these sockets is paramount, as it must balance mechanical efficiency with the ability to accommodate the wearer’s unique anatomical and physiological characteristics [7].

Traditionally, prosthetic sockets have been constructed from bulk polymers or composite materials reinforced with high-strength fibres, such as carbon, glass, and Kevlar [8,9]. While these materials offer significant benefits in terms of durability and strength, they also present challenges related to cost, accessibility, and sustainability [10,11]. These issues are particularly pronounced in less-resourced regions, where the high expense of advanced materials can limit access to quality prosthetic solutions [12]. High-performance laminated composites are often costly due to expensive raw materials and extensive fabrication tooling requirements. Additionally, the resins used in reinforcement infusion processes carry a high environmental impact, and these high-demand materials are not easily accessible worldwide, particularly for global amputees. Thermoset-based polymers, widely used in these composites, are also challenging to recycle after use [13]. Thus, the development of eco-friendly materials with enhanced mechanical, thermal, and tribological properties is highly valued.

In response to these challenges, self-reinforced polymer composites (SRPCs) have emerged as a promising alternative. SRPCs comprise a single polymer matrix that acts as both the reinforcing and matrix phase, providing a unique combination of strength, flexibility, and potential cost benefits [14,15]. Due to material homogeneity, SRPCs provide a high interfacial strength between the matrix and reinforcement, which is difficult to achieve with heterogeneous composites. Another key drawback of CFRP composites is their low failure strain, often leading to catastrophic failure of the reinforcement. In contrast, SRPCs offer a more ductile response with extended elongation at break [16]. It also offers substantial environmental benefits, such as ease of recycling through re-processing by melting, and provides greater flexibility in creating complex shapes.

Given the potential advantages of SRPCs, a thorough examination of their tribological properties is essential [17]. Tribology, the study of friction, wear, and lubrication, is crucial in evaluating how materials perform under various conditions [18,19]. Managing friction between the socket and the residual limb is critical for prosthetic sockets. Excessive friction can lead to discomfort, skin irritation, and pressure-related injuries, such as pressure ulcers and blisters, which can severely impact the quality of life for amputees [20,21]. On the other hand, insufficient friction can result in poor socket retention, leading to instability and reduced prosthesis functionality [22]. Thus, it is essential to understand and regulate friction to ensure comfort and maximise the material performance [23].

Current research on prosthetic socket materials has predominantly focused on traditional polymers like high-density polyethylene (HDPE) and polypropylene (PP) [24,25]. These materials have been extensively studied for their frictional properties and their impact on the socket’s performance [26]. However, SRPCs offer distinct properties that are not yet fully explored in the context of prosthetic applications. Their self-reinforcing structure, which combines high mechanical strength with low weight, could provide benefits in terms of wear resistance and durability that are not apparent in traditional materials [27,28].

To bridge this gap, our study investigates the tribological behaviour of SRPCs, focusing on their friction and wear characteristics under controlled experimental conditions [29]. Although our current tests involve interactions between SRPCs and steel, these results are crucial for understanding the material’s durability and performance [30]. Testing with steel provides a standardised basis for comparison and helps assess the fundamental wear resistance of SRPCs, which is a crucial consideration for their use in prosthetic sockets [31].

Further research will be necessary to evaluate how SRPCs perform in direct contact with human skin or skin-like materials, which will provide more insights into their suitability for real-world prosthetic applications [32]. This study is a foundational step in assessing SRPCs’ potential, paving the way for more comprehensive evaluations of their performance in practical prosthetic scenarios.

Through this tribological analysis of SRPCs, we aim to advance the development of more sustainable and accessible prosthetic solutions. SRPCs offer a promising approach to improving the design and function of prosthetic sockets, ultimately enhancing the quality of life for amputees. Our research seeks to lay the groundwork for future studies to optimise these materials for enhanced comfort and performance in prosthetic applications.

## 2. Materials and Methods

**Figure 1:**
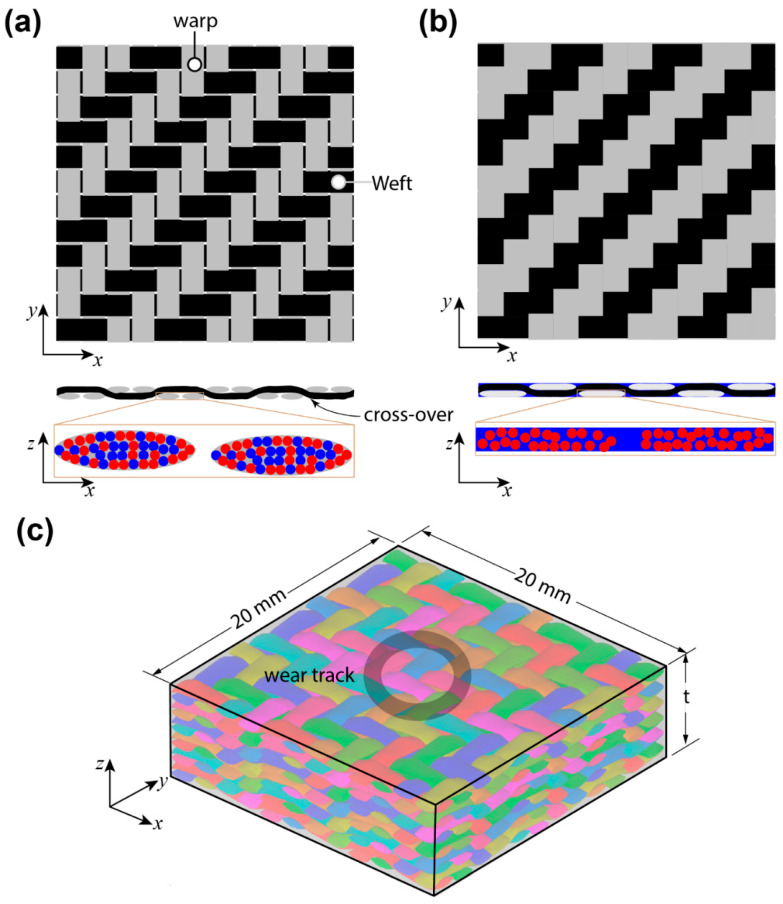
Schematic of the 2/2 twill weave fabric architecture (**a**) as received and (**b**) after vacuum consolidation at elevated temperature. (**c**) Sketch representing the geometrical details of the specimen used for pin-on-disc friction tests.

In this work, all the composite materials were supplied by COMFIL APS^®^, Gjern, Denmark, and monolithic polymers (high-density polyethylene and polypropylene) were received from Bhagwan Mahaveer Viklang Sahayata Samiti (BMVSS—an Indian not-for-profit organisation providing low-cost prostheses in Jaipur, India). The COMFIL APS^®^ uses the commingling process to combine two different types of fibres to produce hybrid yarns. These hybrid yarns were further used in the weaving process to make 2/2 Twill woven fabric for manufacturing the self-reinforced Polymer (srP) composites. The architecture of the received 2/2 Twill woven fabric is shown in Figure 1a. The *x-* and *y*-axes coincide with the weft and warp direction of the 2/2 Twill fabric, whilst the *z*-axis is designated for the fabric thickness. The same type of hybrid yarn is used in both weft and warp directions during the weaving process. In hybrid yarn, one type of fibre functions as a matrix while the remaining fibres produce reinforcement. The matrix fibres are generally made from lower-melting materials than reinforcing fibres. Under heat, only the matrix fibres melt and act as a binder for the reinforcing fibres. Figure 1b shows the architecture of the srP composite after vacuum-assisted consolidation under elevated temperature. It can be seen that the melting of matrix fibre (blue colour) fills voids in the fabric and also encapsulates the reinforcement fibre (red colour).

### 2.1. Manufacturing of Bulk Polymers

The monolithic HDPE sheets were made using HDPE pipes with a 200 mm diameter. The pipes were cut in half and placed inside the oven for 40 min at a temperature of 200 °C. Upon heating, the softened pipes were placed between rigid platens and pressed in the hydraulic press to attain a uniform thickness of 4 mm. A similar approach was followed for PP with an oven temperature of 160 °C for 40 min. This pre-heating and hot-pressing process mirrors the fabrication process of manufacturing prosthetic sockets.

### 2.2. Manufacturing of srP Composite Plates

Table 1 shows the primary constituents and their volume fraction of four types of srPs designated as the srPLA, srPET, srCF, and srGF investigated in this study. The srPLA composite is 100% bio-plastic as it uses low-melting PLA fibres as a matrix, whilst high-temperature PLA fibres serve as reinforcement. The srPET composite is 100% recyclable as it uses low-melting PET fibres as a matrix, whilst high-tenacity PET fibres produce a reinforcement effect. Both srCF and srGF composites have the same low-melting PET fibres as a matrix.

The manufacturer recommends vacuum-assisted consolidation at elevated temperatures to manufacture the srP composites. The dry 2/2 Twill woven fabric size of 400 mm×400 mm was trimmed from the roll. A total of six layers of the trimmed fabric were stacked together and placed inside a vacuum bag. Then, the vacuum bag was transferred to a convective-type autoclave to consolidate the srP composites. We used a purpose-built composite curing oven consisting of eight 500 W ceramic infrared heating elements arranged along its circumference, providing uniform heating throughout the setup. The temperature profile has been programmed into the autoclave’s PID controller. During the consolidation process, 85% vacuum is maintained whilst heating the vacuum bag at the rate of 5 °C/min until it reaches the consolidation temperatures of 165 °C, 200 °C, 220 °C, and 220 °C for srPLA, srPET, srCF, and srGF composite, respectively. At this point, the temperature is held constant for 20 min, followed by cooling down at the rate of 5 °C/min until 100 °C. Then, the srP composite plates were removed from the autoclave. Each type of srP composite plate was manufactured separately as it had different consolidation temperatures.

The fabricated laminates underwent testing to determine their stress, strain, and elastic modulus characteristics. The material properties of these materials are summarised in Table 2. A comprehensive testing procedure and condition description is available elsewhere [3,33].

### 2.3. Friction Testing

The specimens for friction tests were cut from the srP composite plates by using the ${CO}_{2}$ laser engraving machine. Each specimen has the planer dimensions of 20 mm×20 mm for all types of srP composites. The friction tests were conducted by using a ball-on-disc apparatus. The specimen was secured in a rotating disc, and a 6 mm diameter steel ball (surface roughness = 0.07 µm and hardness grade = AISI440C 720HV) was placed on the specimen and loaded against the disc along the *z*-axis. The srP composite specimen was mounted in a rig such that the weft and warp directional tows were perpendicular to the frictional loading. To investigate with different speeds, the rotational speed was set to 30 rpm, 120 rpm, and 240 rpm. The contacting steel ball was cleaned with ethanol before each test. The effect of contact load was examined with three different loads: 2 N, 3 N, and 5 N. Under each testing condition, five samples were tested for reliability, and the deviation of the results was recorded. Thus, the sliding test was performed by rotating the sample against a stationary steel ball at a controlled speed and load for 3600 s.

In addition to the srP composites, the neat PLA and PET matrix specimens were also tested to elucidate the effect of the matrix on their friction response. The neat PET matrix plate (without reinforcing fibres) was prepared using vacuum-assisted consolidation at an elevated temperature of 200 °C.

### 2.4. Surface Morphology Testing

During the sliding test, the wear track surfaces and wear scar on the steel ball were examined under an optical microscope. The Nikon Eclipse LV150N type microscope (Tokyo, Japan) was equipped with an automatic focus which provides the range of depth of the tested specimen. The surface roughness of each srP composite was recorded before the friction tests using the Mitutoyo SJ-400 apparatus (Kanagawa, Japan). The surface roughness of the wear track was also examined after the friction tests.

### 2.5. Microstructural Analysis

The study utilised the Nikon XTH 160Xi X-ray µCT system integrated (Tokyo, Japan) with VGStudio MAX 3.0, incorporating two workstations for image acquisition and reconstruction. To visualise the wear track of the test samples, a parametric algorithm was developed using the MATLAB R2022a (MathWorks, Natick, MA, USA) software.

The total scanning time was set at 10.5 h to ensure accuracy, generating eight frames for each projection to create a single tiff image for the final 3D model. Given the low-density nature of the tested samples, low-energy conditions (50 kV and 50 µA) were employed in the X-ray µCT process to produce a beam spot. Each sample was exposed to X-rays for 1 s in each projection to ensure proper detection while enhancing edge sharpness. The resolution was maintained at 2.5 µm to enhance image quality. Following the acquisition of the X-ray µCT model, it was imported into a 3D Matlab interface for the post-processing of the voxel image.

**Figure 2:**
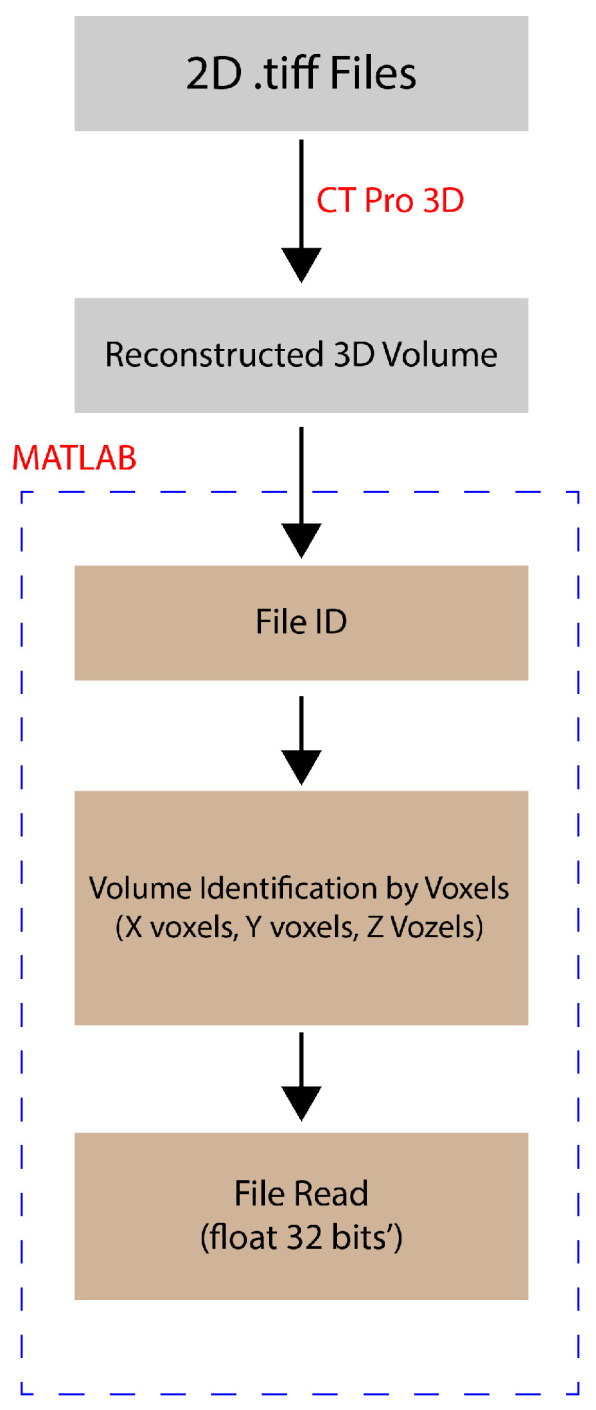
Working principle of X-ray µCT system methodology.

The reconstruction of a 3D model involved combining 3016 2D tiff images produced by the system. Each 2D image was created using eight frames per projection, with the specimen rotation angle step set at 0.12^0^. The 2D slices of the nonwoven model were then sent to a reconstruction workstation, where the CTPro3D software was utilised for the image reconstruction process. For the sake of completion, a flow chart of the post-processing of the image is given in Figure 2; however, a full detail of the post-processing step is given elsewhere [34].

## 3. Results and Discussion

### 3.1. Frictional of Bulk Polymers

**Figure 3:**
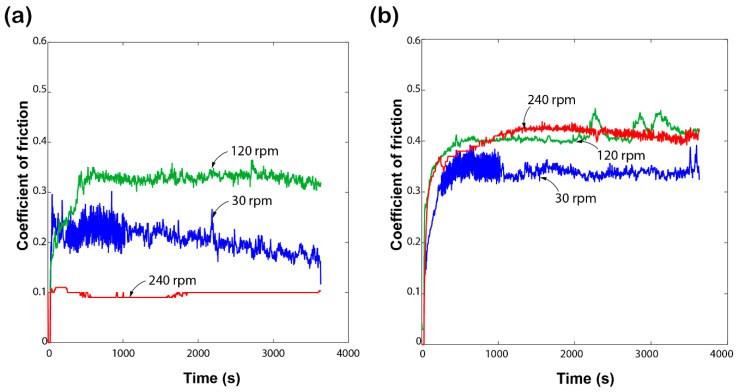
Coefficient of friction response of (**a**) high-density polyethylene and (**b**) polypropylene polymer samples recorded at the constant 5 N load under various rotation speeds.

The frictional response of the HDPE and PP bulk polymers is shown in Figure 3. Both materials were rotated at three different sliding speeds: 30 rpm, 120 rpm, and 240 rpm, with a constant load of 5 N. HDPE exhibits a lower coefficient of friction (COF) at 240 rpm but a higher COF at 120 rpm. Also, we noticed a linear declining trend at the 30 rpm speed after 1000 s. This behaviour suggests that the material is influenced by heat generation, causing the surface to soften and resulting in lower friction at 240 rpm. The response proves that the thermoplastics are highly sensitive to temperature fluctuations, resulting in notable alterations in their tribological characteristics [35].

In contrast to HDPE, PP shows a steady increase in COF with increasing rotation speed. The material maintains a steady response at 30 rpm and 120 rpm, with COF values of 0.35 and 0.4, respectively. However, the COF at 240 rpm exceeds the COF values at 120 rpm, but it starts declining after 1800 s. This indicates that the frictional properties of the bulk polymers are affected by high speed, leading to material damage and deformation, which in turn affects the contact mechanics and friction behaviour [36].

### 3.2. srP Composites with the Same Grade of Polymer

**Figure 4:**
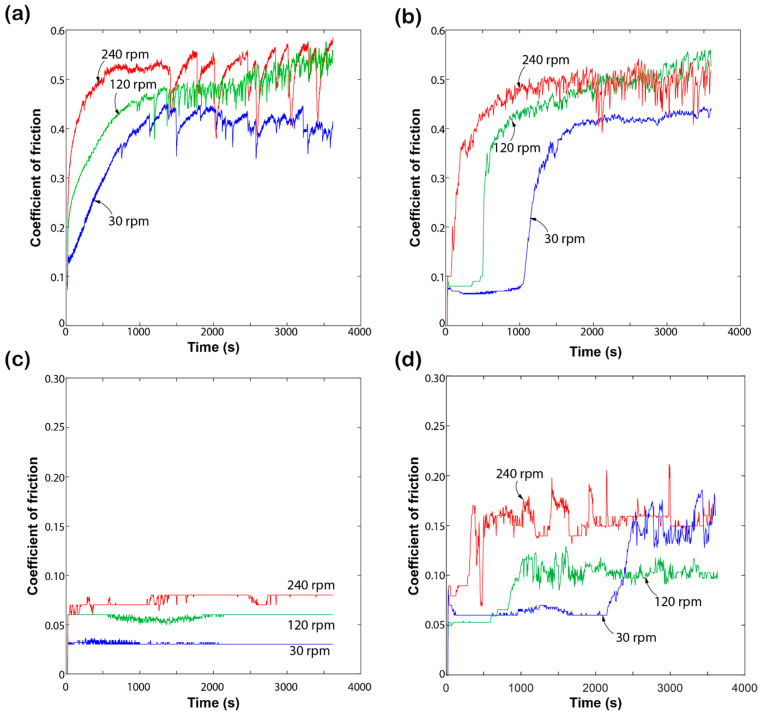
Coefficient of friction (COF) curves of (**a**) neat PLA, (**b**) srPLA, (**c**) neat PET, and (**d**) srPET samples recorded during sliding at a constant load of 5 N under various rotational speeds.

The first series of experiments was conducted on the srP composites (srPLA and srPET) containing matrix and reinforcing fibres made from polymers of the same grade. Figure 4 shows the representative coefficient of friction (COF) curves recorded during sliding against a steel ball for the neat PLA and PET matrix and the srPLA and srPET composite. In all the cases, the specimens are tested at a constant load of 5 N for the rotational speed of 30 rpm, 120 rpm, and 240 rpm. The friction response has three stages in each case: running-in, rising, and steady-state. However, the onset of each stage highly depends on the type of materials and sliding speeds. The neat PLA matrix displays a brief running-in and rising stage followed by a steady state of COF value of 0.48 for the sliding speeds of 120  rpm and 240 rpm (see Figure 4a). However, the neat PLA matrix displays a slower rise in COF values before it reaches the steady-state COF value of 0.45 for the sliding speed of 30 rpm. It can be seen that there is only a mild dependence on the COF values of the neat PLA matrix sliding speed, see Figure 4a. There is a clear demarcation between the run-in and rising stage observed for the srPLA composites, see Figure 4b. The rising stage highly depends on the sliding speeds. The reinforcing effect of PLA fibres in the srPLA composite tends to delay the onset of the rising stage compared to the neat PLA matrix (Figure 4a,b). However, the steady-state COF values between the neat PLA matrix and srPLA composites seem to be approximately similar for each sliding speed reported in Figure 4a,b.

Figure 4c,d shows the COF response of the neat PET matrix and srPET composite tested at a constant speed of 5 N under the sliding speeds of 30 rpm, 120 rpm, and 240 rpm. The COF response of the neat PET matrix shows a brief running-in period followed by a steady-state stage (Figure 4c). The steady-state COF values of the neat PET matrix increase with the increase in sliding speeds. In contrast, the srPET composite displays all three stages in their friction response (Figure 4d). The rising stage highly depends on the sliding speeds, as observed in the srPLA composites. It can be seen that the srPET composite steady-state COF values are unaffected by the sliding speeds (Figure 4d). However, the steady-state COF values of the srPET composite are higher than the neat PET matrix for each sliding speed reported in Figure 4c,d.

Embedding the same grade of fibre reinforcement in the neat thermoplastic polymer matrix influences the friction behaviour of the srP composites compared to the neat polymer. The primary influence is the delay in the onset of the rising stage COF values as a function of the sliding speeds.

It has also been shown that the steady-state COF values have fewer variations for sliding speeds above 120 rpm for a constant load of 5 N. However, the magnitude of the steady-state COF values depends upon the type of polymer grade. For example, the steady-state COF values of srPLA are three times higher than the srPET composite tested at 240 rpm at a constant load of 5 N.

The behaviour of the polymer composites aligns with the ductility of their respective composite materials. Table 2 shows that the srPET exhibits more ductility than the srPLA composite. The srPET’s strain of 20% aligns with its frictional response, indicating a longer time for fibre pull-out at lower speeds. The process of fibre pull-out occurs when stress is applied to the composite, causing the individual fibres to detach from the surrounding matrix material. The relationship between ductility and friction remains consistent for srPLA, as it demonstrates a longer time for fibre pull-out at low speeds and a shorter time at high speeds.

### 3.3. srP Composites with Dissimilar Materials

**Figure 5:**
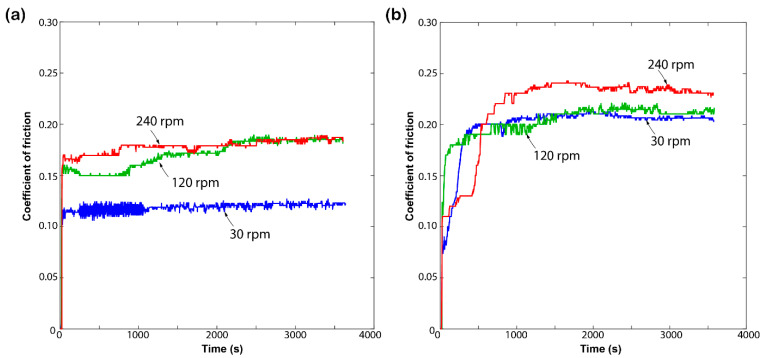
Coefficient of friction (COF) curves of (**a**) srCF and (**b**) srGF composite samples recorded during sliding at a constant load of 5 N under various rotational speeds.

The second series of wear experiments was carried out on the srCF and srGF composites with dissimilar matrix and fibre reinforcements. For comparison purposes, we present the representative coefficient of friction (COF) curves recorded during sliding against a steel ball for srCF and srGF tested at a constant load of 5 N for the rotational speed of 30 rpm, 120 rpm, and 240 rpm in Figure 5. The srCF display a brief running-in stage followed by the steady-state COF response in Figure 5a. The srGF composite displays a brief running-in and rising stage followed by the steady-state COF response in Figure 5b. In both cases, the steady-state COF response increases with the increase in the sliding speeds.

The frictional behaviour of the dissimilar polymeric composites is consistent with the brittle nature of the material. Table 2 illustrates that SrCF and SrGF exhibit lower strain, at 2.24% and 2.17%, respectively. As a result of the elastic–brittle failure of these composites, fibre pull-out occurs more rapidly. A tribological study involving different polymeric composites reinforced with carbon fibres has shown that the formation of a transfer film influences friction levels. The transfer film becomes unstable within the glass-transition region, leading to increased friction [37].

In summary, the srP composite having a PET matrix reinforced with PET, carbon, and glass fibre increases the steady-state COF values compared to the neat PET counterparts. The glass fibre reinforcement seems to have a higher increase in the steady-state COF values of the srP composites than the PET and carbon fibre reinforcement. However, the steady-state COF values of the srP composite having PLA matrix and fibres are approximately the same as their matrix counterparts. In all the cases, the sliding speeds influence the steady-state COF values. It is also observed that there is a significant amplitude fluctuation of the steady-state COF values in all the cases. As discussed later, this is due to the vibration of the ball as it slides over the cross-over junction of the 2/2 Twill weave of the srP composite, causing the stick–slip phenomenon.

### 3.4. Effect of Load

**Figure 6:**
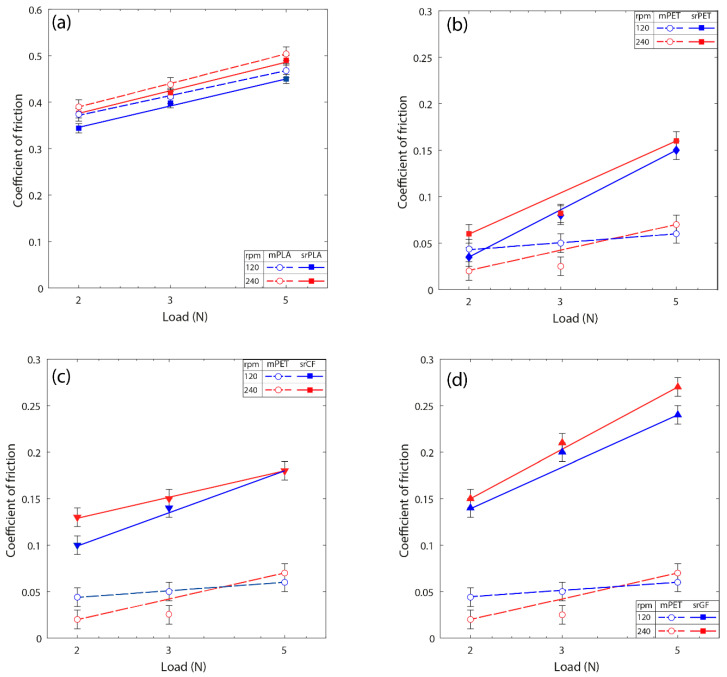
Average coefficient of friction as a function of contact loads at 120 and 240 rpm: (**a**) srPLA, (**b**) srPET, (**c**) srCF, and (**d**) srGF. In each case, the coefficient of friction values for the neat matrix is also compared.

The averaged steady-state COF values for all the srP composites were calculated and presented in Figure 6 as a function of contact loads at the selected sliding speeds of 120 rpm and 240 rpm. This figure also includes the data for the neat PLA and PET matrix for comparison purposes. The error bar indicates the confidence in measurement from five tests conducted for each condition and specimen. In all the cases, the sliding speeds and contact loads influence the steady-state COF values. The most substantial influence arises from the contact loads rather than the sliding speeds. The neat PLA and srPLA composites have higher COF values than those of the srP composites, which have PET matrices reinforced with carbon, glass, and PET fibres. The increase in the steady-state COF values of the srPLA composite is marginal compared to the neat PLA matrix in all conditions. However, there is a substantial increase in the steady-state COF values in the srP composites with PET matrix reinforced with carbon, glass, and PET fibres compared to their matrix counterparts.

### 3.5. Surface Property Observation

**Figure 7:**
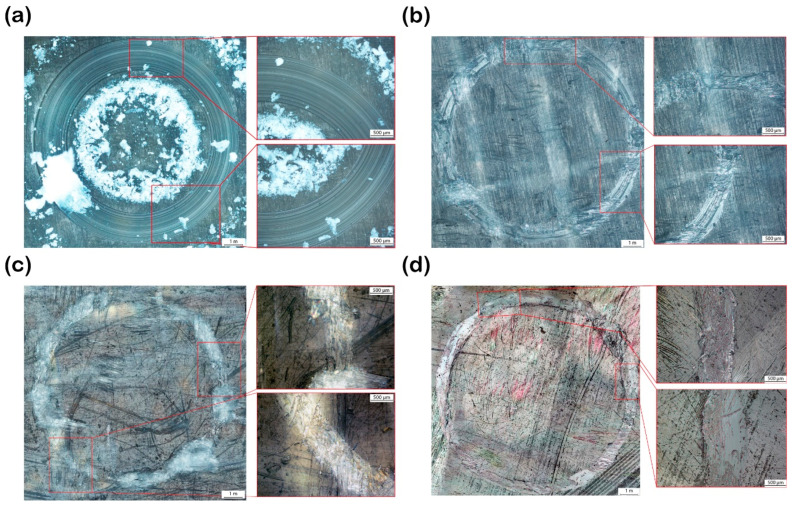
Microscopic image showing the wear track of (**a**) srPLA; (**b**) srPET; (**c**) srGlass Fibre; (**d**) srCarbon Fibre.

Approximately 60 images were captured around the wear surface using the microscope to produce the full-field view of the wear surface of the srP composite. For comparison purposes, we present the micrographs for the srP composites tested at a constant load of 5 N for a sliding speed of 240 rpm. Figure 7a shows the full-field micrograph for the srPLA composite specimen. The wear tracks consist of lines along the sliding direction, confirming the longitudinal ploughing mechanism for material removal during friction testing. The material debris consists of pulverised fragments of both matrix and fibres. It can be seen that the material debris is collected on either side of the wear track. The inset in Figure 7 also confirms that the fibre thinning mechanism is operating during friction testing. Figure 7b shows the full-field micrograph of the wear surface for the srPET composites. In some areas of the wear track, we observe lines confirming that the longitudinal ploughing mechanism is operating. The reinforcement fibres are pulled out when the ball slides over the yarn cross-over junction of the 2/2 Twill weave (refer to Figure 1a). Either side of the cross-over junction represents the polished wear surface. This indicates that the ball jumps over at a cross-over junction, causing high amplitude fluctuation in the measured friction response in Figure 4d. Unlike the srPLA composite, no obvious material debris is seen on either side of the wear track of the srPET composite. As discussed later, the material debris stick to the ball’s surface.

The full-field micrograph of the wear surface for the srCF composites is displayed in Figure 7d. The material debris in the form of pulverised fibre fragments is seen on either side of the wear surface. Again, the fibres are being pulled out of the composites at the yarn cross-over junction. Figure 7c shows the full-field micrograph of the wear surface for the srGF composites. The polished surface of the wear track is visible. Not much material debris is observed on either side of the wear track. Likewise, in the srPET and srCF composite, the reinforcement fibres are pulled out of the composite at the yarn cross-over junction in the srGF composite.

**Figure 8:**
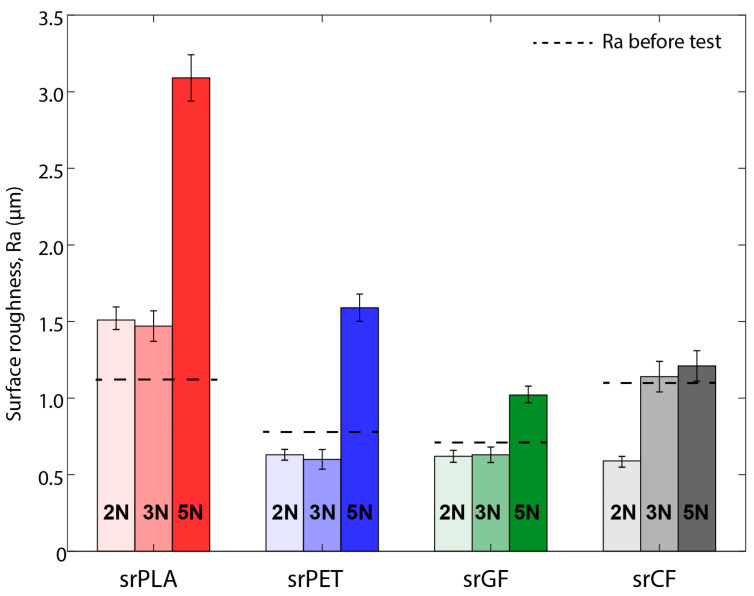
Surface roughness at 240 rpm for SrPLA, SrPET, SrGlass fibre, SrCarbon fibre.

**Figure 9:**
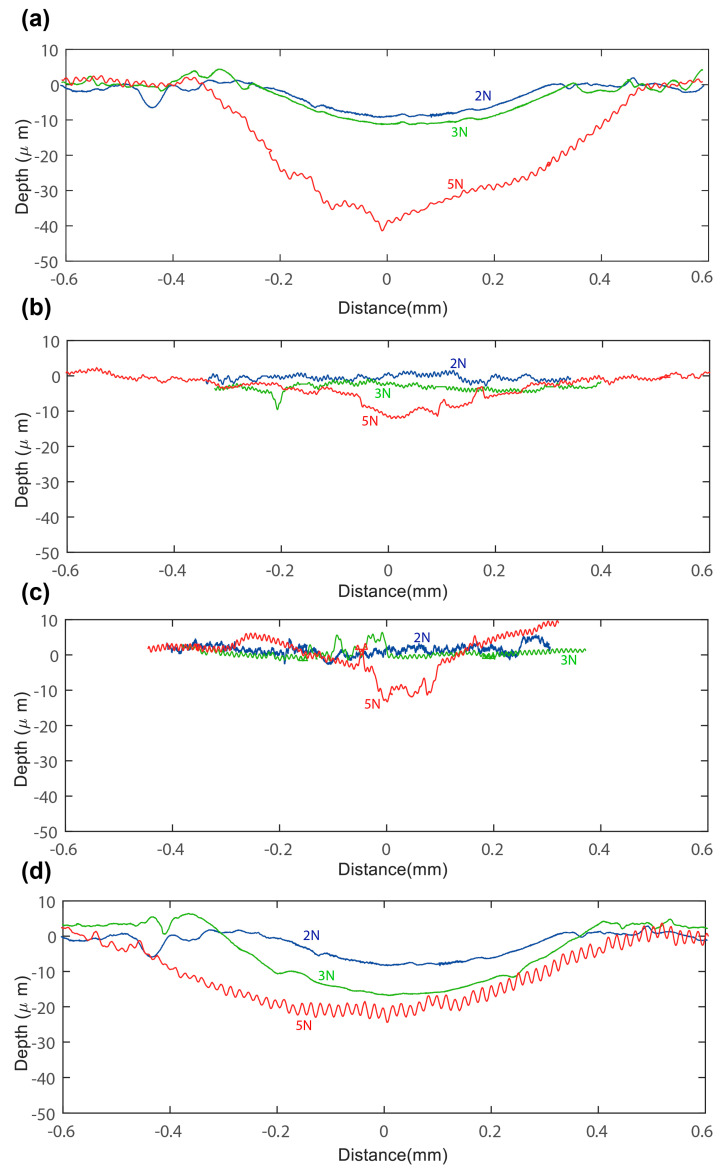
Wear track at 240 rpm in different load conditions: (**a**) SrPLA, (**b**) SrPET, (**c**) SrGF, and (**d**) SrCF.

The surface roughness of the SRP materials and the wear cross-sectional area are displayed in Figure 8 and Figure 9. The first series of experiments was conducted before the test, and the second series was conducted after the test under various conditions. The error bar in Figure 8 represents the range of surface roughness values from five experiments conducted for each material. The influence of the conditions was highly noticeable in the surface roughness of these composites. The increase in the surface roughness of SrPLA indicates that the material underwent mechanical wear, with the extent of the increase being proportional to the applied load. The srPLA shows a high $R_{a}$ of 3.1 μm at 5 N load compared to the 1.1 μm before the test. Similarly, the $R_{a}$ of srPET, srGF, and srCF were increased to 1.6 μm, 1.0 μm, and 1.15 μm from 0.7 μm, 0.6 μm, and 1.1 μm before the test. Upon comparing the $R_{a}$, higher differences were noticed after the 5 N contact load for srPLA, followed by srPET, srGF, and srCF. These changes in surface roughness show that srPLA experienced high wear and rough surface under the applied mechanical stress.

The wear depth at this condition was investigated and presented in Figure 9. The negative value in the plot illustrates the material loss. In contrast, the position value suggests the material build-up or deposition after the test. Despite the high surface roughness of srPLA, it exhibits a wear depth of −40 μm. The srPET, srCF, and srGF show less wear depth of −11 μm, −12 μm, and −24 μm, indicating less material loss than srPLA. Though srPET, srGF, and srCF have the same matrix, the surface roughness and wear depth differences among these materials are highly influenced by the properties of the reinforcing fibres. High material depositions were noticed in srGF, where the material was pushed out of the track during the test, which built up the material. The elastic–brittle nature of carbon fibre is prone to less wear depth with low surface roughness. It was previously observed that the specific wear rate increased as the applied force increased for the polymer composites [38,39].

Computed Tomography (CT) scanning was utilised to investigate the wear effects on polymer composite specimens post-frictional testing. The analysis revealed distinct wear patterns and structural changes, particularly in the PLA- and PET-based materials, while minimal wear was observed in the carbon fibre (CF) and glass fibre (GF) specimens.

**Figure 10:**
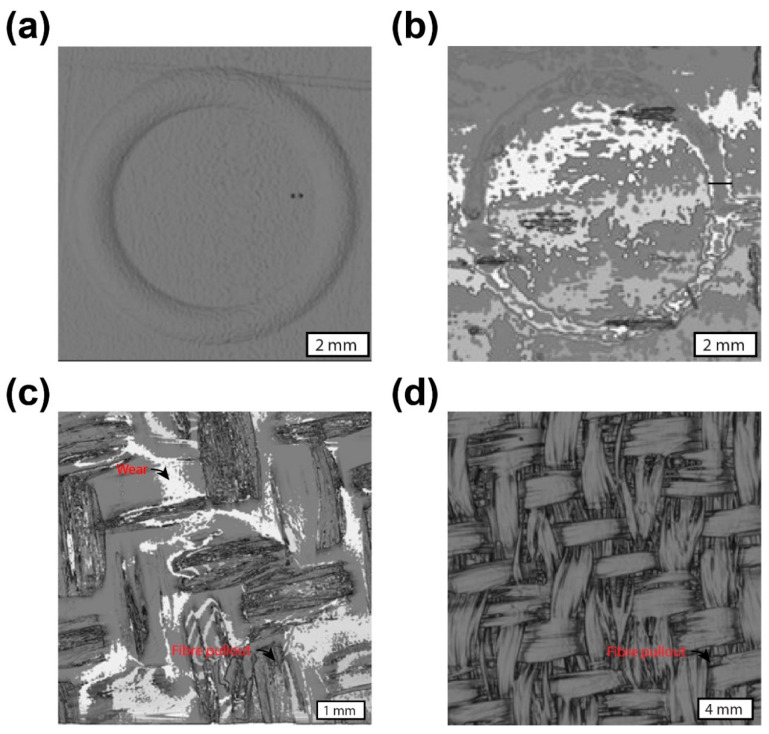
CT scans revealing wear effects on polymer composite specimens: (**a**) SrPLA, (**b**) SrPET, (**c**) SrGF, and (**d**) SrCF (each specimen size is 20 mm × 20 mm).

The CT scans demonstrated prominent wear effects on the PLA-based specimens, indicating significant material degradation and surface alterations. The visual indications of wear were corroborated by CT imaging, highlighting the susceptibility of the PLA composites to wear-induced damage. Similarly, the PET-based specimens exhibited noticeable wear effects under CT imaging, albeit to a lesser extent than PLA. Structural changes and material loss were observed, suggesting the moderate wear resistance of PET composites. Remarkably, the CF and GF specimens displayed minimal wear effects under CT scanning, despite the visual indications of wear marks. The CT images revealed virtually no trace of wear or structural damage in these specimens, indicating robust wear resistance and structural integrity. The discrepancy between the visual wear marks and CT imaging findings for the CF and GF specimens suggests that surface wear may not necessarily translate to structural damage, emphasising the importance of advanced imaging techniques for a comprehensive wear analysis. The results of the CT scan are shown in Figure 10.

The CT scan analysis aligns with the optical observations, highlighting the differential wear behaviour among the polymer composite specimens. The pronounced wear effects observed in the PLA- and PET-based materials underscore their susceptibility to friction-induced degradation, whereas the CF and GF specimens exhibit superior wear resistance and resilience. These findings underscore the critical role of material composition in determining wear resistance and durability. Future research may explore underlying mechanisms governing wear behaviour in polymer composites to enhance their performance and longevity in various applications.

### 3.6. Material Assessment of Steel Ball

**Figure 11:**
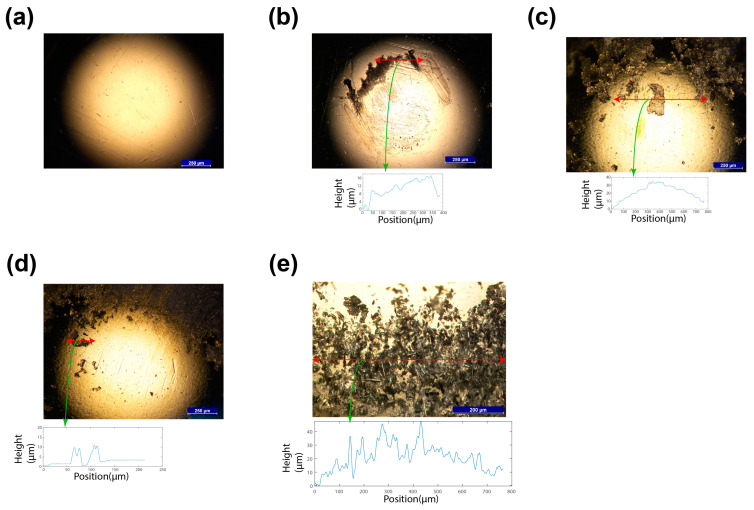
The contacting ball under 5 N at 240 rpm and the depth profile of the track: (**a**) the steel ball before testing the steel ball’s surface tested against the (**b**) srPLA, (**c**) srPET, (**d**) srGF, and (**e**) srCF composites.

Prior to and after the friction testing, the steel ball was subjected to optical microscopy investigation. Figure 11 illustrates the micrographs of the steel ball’s sliding surface before testing and after testing against the srPLA, srPET, srCF, and srPET composite at a constant load of 5 N with a sliding speed of 240 rpm. In all the cases, the debris adhered to the ball surface for all types of srP composites. The debris consists of pulverised fragments of matrix as well as the fibres. This confirms that the reinforcement fibres are being pulled out at the wear sites during friction testing. These images are evidence that the contact between the sliding surfaces was non-uniform due to the sticking of debris between the ball and the wear surface of the specimen. This may be the reason for increasing the steady-state COF values of the srP composite compared to their matrix counterparts. The matrix offers a smooth surface to slide against the ball. However, the srP composites have fibre fragments at the contact surface juncture providing a texture; thus, it leads to an increase in the COF values.

A greater extent of fibre pull-out was observed for SrCF in Figure 11e. We observed from the steel ball that the load exerted on the material pushes it, resulting in reduced adhesion between the centre of the ball and the fibres, while the material accumulates on the sides of the ball. A thin film transfer may develop between the thermoplastic materials and the metal counterfaces. Friction-induced heat causes the localised softening or distortion of the thermoplastic matrices when exposed to frictional forces. This softening can facilitate the formation of a lubricating layer between the fibres and the contacting surface. Polymers are recognised for their high strength–modulus ratios and low surface energies, which contribute to wear reduction by promoting significant elastic stresses and facilitating the transfer of protective films to high-energy surfaces. However, at elevated interface temperatures, plastic deformation occurs, compromising the soft surface and resulting in substantial damage [40].

### 3.7. Comparison of srP with Other Polymers

**Figure 12:**
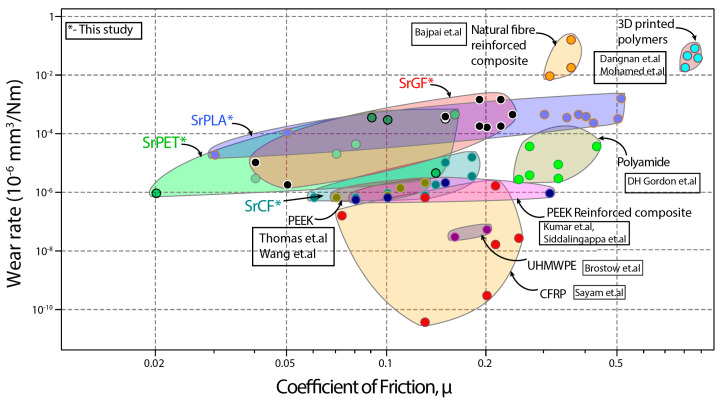
Comparison of wear rate and coefficient of friction of self-reinforced composites with other polymer materials [38,41,42,43,44,45,46,47,48,49].

The wear rate and coefficient of friction for a range of polymers and self-reinforced composites are displayed in Figure 12. The SrP composites exhibit a wide range from low COF to high COF. However, the wear rate of the SrP composites is comparatively higher than most of the polymers. It can be observed that the carbon fibre with a thermoset-based matrix shows a lower wear rate than self-reinforced carbon fibre. The usage of additive manufacturing is increasing in the prosthetic industry, where 3D-printed polymers show relatively high friction compared to srPET with a lower wear rate.

The observed frictional responses of SrPCs, particularly the differential behaviour between materials with similar and dissimilar matrices and reinforcement fibres, offer implications for prosthetic socket design. For instance, the delay in the onset of the rising stage of the coefficient of friction (COF) with increasing sliding speeds in SRPCs indicates potential benefits in reducing friction-induced injuries, as it allows for a smoother transition to steady-state friction. Furthermore, despite visual wear marks, the minimal wear effects observed in the carbon fibre (CF) and glass fibre (GF) specimens underscore the importance of selecting materials with robust wear resistance for prosthetic sockets.

By extrapolating our findings, prosthetic designers can optimise skin–socket interface interactions by leveraging materials with tailored frictional properties, thereby reducing the risk of pressure ulcers, blisters, and discomfort for amputees. Self-reinforced polymer composite (SRPC) development presents a promising avenue for enhancing prosthetic socket durability and wearer comfort. Integrating these friction results into prosthetic socket design underscores the interdisciplinary nature of prosthetic research, where insights from tribology, materials science, and biomedical engineering converge to improve amputee quality of life. Continued investigation into the tribological aspects of prosthetic materials will play a pivotal role in advancing prosthetic technology. Understanding material friction properties is crucial for optimising prosthetic socket functionality and mitigating skin trauma and mobility limitations.

Frequently, prosthetic sockets were worn with a suspension system, either a silicone liner or cotton socks. The connection between the prosthetic socket and the suspension system is crucial for socket comfort. The coefficient of friction (CoF) of socket materials plays a crucial role in determining the quality of this connection. This study only investigates the new prosthetic materials under dry friction conditions. Further work is needed to understand the interaction between these newly developed SRPCs along with suspension system.

## 4. Conclusions

This study provides a comprehensive analysis of self-reinforced polymer composites’(SRPCs) frictional and wear characteristics, offering valuable insights into their potential for prosthetic socket applications. The results demonstrate distinct frictional behaviours among different materials, with traditional polymers like HDPE and PP exhibiting lower friction coefficients due to heat generation and surface softening. In contrast, self-reinforced composites, including SrPLA, SrPET, SrGF, and SrCF, display a more comprehensive range of friction coefficients and wear rates, highlighting the need to consider both material properties and application-specific factors during selection carefully.

One of the critical advantages of SRPCs is their manufacturing efficiency. These composites allow for reduced production time compared to conventional resin infusion methods, making them a cost-effective solution for prosthetic socket fabrication. Additionally, materials like SrPLA and SrPET offer significant sustainability benefits, such as biodegradability and recyclability, which enhance their appeal for environmentally friendly prosthetic manufacturing. These factors position SRPCs as viable alternatives to traditional materials, especially in less-resourced regions where affordability and accessibility are critical concerns.

The tribological performance of these composites was further influenced by their interaction with steel, which generated a film transfer on the steel ball counterface, improving both frictional stability and wear resistance. However, the brittleness of SrCF and SrGF led to fibre pull-out and earlier wear compared to the more ductile SrPLA and SrPET materials, which performed better under varying loads and rotational speeds. Despite the higher friction coefficients exhibited by certain SRPCs, their overall performance suggests that with proper optimisation, they could significantly enhance the comfort and durability of prosthetic sockets.

The findings of this study underscore the importance of tribological analysis in evaluating new materials for prosthetic applications. By integrating insights from tribology, materials science, and biomedical engineering, self-reinforced composites show considerable promise in advancing prosthetic technology. More controlled experiments are necessary to understand how SRPs interact with silicone liners’ wet or deformable mating surfaces. This is important for simulating the interaction between human skin and silicone liners. Particular attention should be given to ambient and vacuum conditions to replicate vacuum-assisted socket suspension, as suction suspension relies on direct contact between the liner and the socket wall.

While further research is necessary, particularly in evaluating the interaction between SRPCs and skin, this study lays the groundwork for developing more functional and comfortable prosthetic sockets that address the mobility limitations of amputees. Through continued innovation, SRPCs hold the potential to improve the quality of life for individuals with limb loss and contribute to more sustainable, accessible prosthetic solutions.

## Figures and Tables

**Table 1:** Primary constituent of the commingled yarns that were used to manufacture the self-reinforced polymer composite in this study.

Designation	Matrix	Reinforcement	Volume Fraction		$\mathrm{Tex}\;(g/m^{2})$
			$\mathrm{Matrix}\;(v_{m})$	$\mathrm{Fibre}\;(v_{f})$	
srPLA	LPLA	HPLA	50	50	360
srPET	LPET	HPET	49	51	440
srCF		Carbon	46	54	1500
srGF		Glass	43	57	1570

**Table 2:** Material properties of the standard prosthetic socket material and self-reinforced polymer composites used in this study.

Parameters	srPLA	srPET	srCF	srGF	PP	HDPE
Young’s Modulus $\left(E_{1},\;\mathrm{GPa}\right)$	3.85 ± 0.52	4.45 ± 0.31	28.3 ± 0.28	21.05 ± 1.15	0.3 ± 0.04	0.86 ± 0.07
Young’s Modulus $\left(E_{2},\;\mathrm{GPa}\right)$	3.7 ± 0.39	4.35 ± 0.25	22.6 ± 0.7	16.8 ± 3.2	-	-
Strain to failure $\left(\varepsilon_{1}^{f},\;\%\right)$	1.6 ± 0.1	19.5 ± 0.75	2.24 ± 0.05	2.17 ± 0.13	>50%	49 ± 0.96
Strain to failure $\left(\varepsilon_{2}^{f},\;\%\right)$	1.8 ± 0.15	18.7 ± 0.56	2.05 ± 0.05	2.2 ± 0.1	-	-
Failure Stress $\left(\sigma_{1}^{f},\;M\mathrm{Pa}\right)$	40 ± 0.5	127 ± 4	358.5 ± 7.77	258 ± 23	7.4 ± 0.48	17.17 ± 0.22
Failure Stress $\left(\sigma_{2}^{f},\;M\mathrm{Pa}\right)$	43 ± 0.2	132 ± 5	223.5 ± 2.12	242 ± 8	-	-

## Data Availability

The original contributions presented in the study are included in the article, further inquiries can be directed to the corresponding author.
